# ﻿*Microtoenawawushanensis* (Lamiaceae, Lamioideae): A new species from Sichuan, China

**DOI:** 10.3897/phytokeys.250.139362

**Published:** 2024-12-30

**Authors:** Xue-Xue Wu, Yan Wang, Yan-Yi Chen, Qiang Wang

**Affiliations:** 1 State Key Laboratory of Plant Diversity and Specialty Crops, Institute of Botany, Chinese Academy of Sciences, Beijing 100093, China; 2 National Botanical Garden, Institute of Botany, Chinese Academy of Sciences, Beijing 100093, China; 3 College of Life Sciences, University of Chinese Academy of Sciences, Beijing 100049, China

**Keywords:** Lamiaceae, *Microtoena*, new taxon, taxonomy

## Abstract

*Microtoenawawushanensis*, a new species from Sichuan, China, is described and illustrated here. The new species is closely related to *M.moupinensis* and *M.prainiana*, but differs distinctly from both in leaf, calyx and bract morphology. It is further distinguished by its highly variable and unstable calyx tooth ratio (1.36–2.13), red-marked lateral lobes on the lower corolla and filaments that are barbate at both the upper and basal parts, with nearly imperceptible hairs in the middle section. Phylogenetic analyses, based on 81 coding regions of the chloroplast genome, suggest that *M.wawushanensis* belongs to sect. Delavayana and is sister to a clade formed by *M.urticifolia*, *M.prainiana* and *M.megacalyx*.

## ﻿Introduction

*Microtoena* Prain was established by David Prain in 1889 (Prain 1889). Recent studies, based on various chloroplast and nuclear gene fragments, consistently show that *Microtoena* belongs to the tribe Pogostemoneae Briq. of the Subfam. Lamioideae Harley within the Lamiaceae Martinov, closely related to *Pogostemon* Desf. and *Anisomeles* R. Br. (Scheen et al. 2010; Bendiksby et al. 2011; Li et al. 2016; Yao et al. 2016; Zhao et al. 2021a, b). Based on the monograph of *Microtoena* (Wang 2018), there are 19 species of *Microtoena* globally, with 18 species widely distributed across China, ranging from Gansu to Hainan. Phylogenetic analyses of 11 species within the genus, based on three chloroplast markers, revealed that *Microtoena* can be divided into two distinct sections, sect. Microtoena and sect. Delavayana, which are also supported by morphological evidence (Wang 2018).

Biodiversity loss and species extinction are being accelerated globally, largely driven by human-induced population growth and climate change (Huang et al. 2023; Mi et al. 2023). In an effort to mitigate biodiversity degradation, the Chinese government has undertaken numerous initiatives (Mi et al. 2021; Huang et al. 2023; Mi et al. 2023; Ren and Antonelli 2023), amongst which the establishment of national botanical gardens stands out as a significant achievement (Huang et al. 2023; Ren and Antonelli 2023). The Qinghai-Tibetan Plateau is a natural habitat for rare plants and a gene pool of plateau life, recognised globally as a biodiversity hotspot and receiving significant attention (Sun et al. 2017; Lu et al. 2018; Liu et al. 2021; Mi et al. 2021; Zhang et al. 2023). It has also become a focal point of national botanical garden initiatives. As members of the first national botanical garden of China (Ren and Antonelli 2023), we are engaged in the ongoing compilation of the Lamiaceae volume for the Flora of Pan-Himalaya (FPH) project (Wang and Hong 2022). We made an expedition to Sichuan Province last year and encountered a population in the Wawushan Nature Reserve, Meishan City, which could be distinctly differentiated from previously described *Microtoena* species, based on characteristics such as corolla colour, clear presence or near imperceptibility of hair on the middle parts of filaments and calyx morphology. After a comprehensive review of specimens, literature and incorporating detailed morphological and molecular features, we confirmed that this plant represents a new species within the *Microtoena* genus in China.

## ﻿Materials and methods

### ﻿Taxa sampling

We selected 13 individuals from the genus *Microtoena* for our analyses, representing nine distinct species. These taxa include representatives from the two sections currently recognised in *Microtoena* (Wang 2018), as well as those that are morphologically similar to newly-described species. For our outgroup, we used *Pogostemon*, *Anisomeles* and *Craniotome* Rchb., following a previous study (Yao et al. 2016; Wang 2018; Yuan et al. 2022). All ingroup samples were newly sequenced for this study, totalling 13 samples, while sequences for the three outgroup species were obtained from public datasets (see Table 1). Amongst the materials used in our research, three samples consisted of silica gel-dried fresh leaves collected from the field and 10 were sourced from herbarium specimens deposited in the herbarium of the Institute of Botany, Chinese Academy of Sciences (PE), with permission. Detailed information regarding the taxa used in this study can be found in Table 1.

**Table 1.** Voucher information of samples for phylogenetic analyses and GenBank accession numbers.

	Taxon	Voucher	Sample Form	GenBank accession numbers
1	*Microtoenaurticifolia* Hemsl._89	Z.P.Jian et al. *31157* (PE)	specimen leaves	PQ664497
2	*Microtoenaurticifolia* Hemsl._90	Z.P.Jian et al. *31157* (PE)	specimen leaves	PQ664498
3	*Microtoenaprainiana* Diels_83	Q.Wang *H12079* (PE)	specimen leaves	PQ664495
4	*Microtoenaprainiana* Diels_W24-19	Q.Wang *H12079* (PE)	specimen leaves	PQ664500
5	*Microtoenamoupinensis* (Franch.) Prain_78	D.Y.Hong et al. *H12056* (PE)	specimen leaves	PQ664493
6	*Microtoenamoupinensis* (Franch.) Prain_86	K.J.Guan et al. *3219* (PE)	specimen leaves	PQ664496
7	*Microtoena megacalyx_* C.Y.Wu_*72*	Y.M. Shui *003011* (PE)	specimen leaves	PQ664492
8	*Microtoenarobusta* Hemsl._W24-16	J.Q.Fu *2756* (PE)	specimen leaves	PQ664499
9	*Microtoenadelavayi* Prain_W037	Q.B.Gong *CPG47960* (PE)	silica gel-dried leaves	PQ664501
10	*Microtoenadelavayi* Prain_W038	Q.B.Gong *CPG46899* (PE)	silica gel-dried leaves	PQ664502
11	*Microtoenainsuavis* (Hance) Prain ex Briq._71	X.Y.Liu *24836* (PE)	specimen leaves	PQ664491
12	*Microtoenapatchoulii* (C.B. Clarke ex Hook. f.) C.Y. Wu & S.J. Hsuan_82	Q.Wang *H&W09011* (PE)	specimen leaves	PQ664494
13	*Microtoenawawushanensis* Xue X. Wu & Qiang Wang	X. X. Wu et al. *WXX23001* (PE)	silica gel-dried leaves	PQ664503
14	*Pogostemoncablin* (Blanco) Benth.	–	–	MF445415
15	*Anisomelesindica* (L.) Kuntze	–	–	NC_46781
16	*Craniotomefurcata* (Link) Kuntze	–	–	NC_54194

### ﻿DNA extraction, sequencing, genome assembly

DNA extractions were performed using a modified cetyltrimethylammonium bromide (CTAB) method as outlined by Doyle and Doyle (1987) for specimens or with the Magnetic Plant Genomic DNA Kit from TIANGEN BIOTECH (Beijing) Co., Ltd. for silica gel-dried samples. Library preparation and whole genome sequencing (WGS) were carried out at Novogene Bioinformatics Technology Co., Ltd. in Beijing, China. Initially, the genomic DNA samples were fragmented. Following this, the fragments underwent end polishing, A-tailing, ligation with full-length sequencing adapters and PCR amplification. After quality assessment and quantification, the eligible libraries were sequenced using either the DNBSEQ-T7 or Illumina NovaSeq 6000 platform according to the PE150 strategy.

In this study, we utilised GetOrganelle software (Jin et al. 2020) to assemble the chloroplast genetic sequencing data for the target taxa listed in Table 1. The assembly results were saved as a GFA file and later imported into Bandage (Wick et al. 2015) for visualisation and verification. The assembled genomes were annotated using the Plastid Genome Annotator (Qu et al. 2019), with a re-annotated *Craniotomefurcata* (Link) Kuntze (NC_054194) from GenBank serving as the reference.

### ﻿Molecular phylogenetic analyses

Single gene matrices were generated using Geneious Prime 2022. The matrices for each of the 16 plastomes were aligned using MAFFT v.7.3.1 (Katoh and Standley 2013) in PhyloSuite v.1.2.2 (Zhang et al. 2020). Loci with abnormal high variation were removed using the default parameters of Gblocks (Castresana 2000). The final coding regions (CDS) matrices from the 16 taxa were manually checked using AliView v.1.26 (Larsson 2014). Subsequently, all individual CDS matrices were concatenated into a single supermatrix using PhyloSuite v.1.2.2 (Zhang et al. 2020).

Phylogenetic trees were constructed using Bayesian Inference (BI) methods and Maximum Likelihood (ML) methods. The BI analysis was performed with MrBayes v.3.2.7a (Ronquist et al. 2012), using the MCMC (Markov Chain Monte Carlo) algorithm run for 1,000,000 generations, saving a tree every 1,000 generations. The first 25% of the trees were discarded. The analysis was stopped when effective sample sizes (ESS) exceeded 200 and the average standard deviation (SD) of split frequencies was below 0.01. ML analysis was performed using RAxML v.8.2.12 (Stamatakis 2014) with the GTRGAMMA model and 1000 bootstrap replicates.

### ﻿Morphological and taxonomy study

Apart from the new species, all morphological samples of the genus *Microtoena* were obtained from the Herbarium PE (Table 1). Photographs of *Microtoena* morphologies were taken using an Olympus EM5 Mark III and a Nikon D7500 digital camera. Details of the filaments of the *Microtoena* were captured using a Leica M205C stereoscopic microscope. Morphological data were collected by measuring specimens indoors with a measuring tape and analysing photographed specimens using MATO software (Liu et al. 2023).

We used analytical indices for the bracts and calyx introduced by Wang (2018): BR (bract ratio) = BW (bract width) / BL (bract length), CLA (calyx length in anthesis), SR (split ratio) = OTL (average of ordinary tooth length of calyx) / CL (calyx length) and TR (tooth ratio) = LLT (length of the longest tooth) / OTL (average of ordinary tooth length of calyx).

## ﻿Results and discussion

### ﻿Sequence characterisation

Our ingroup samples were sequenced, yielding a range of 42.12G to 156.18G of raw data, with an average of 79.9G (Suppl. material 1). All of the 13 samples had complete chloroplast genomes assembled. The lengths of *Microtoena* plastomes in this research range from 152742 bp (*M.insuavis* (Hance) Prain ex Briq) to 153004 bp (*M.prainiana* Diels). All individuals of 81 CDS matrices (i.e. CP81) were concatenated into one single 68346 bp-long supermatrix.

### ﻿Molecular phylogeny results

**Figure 1.**
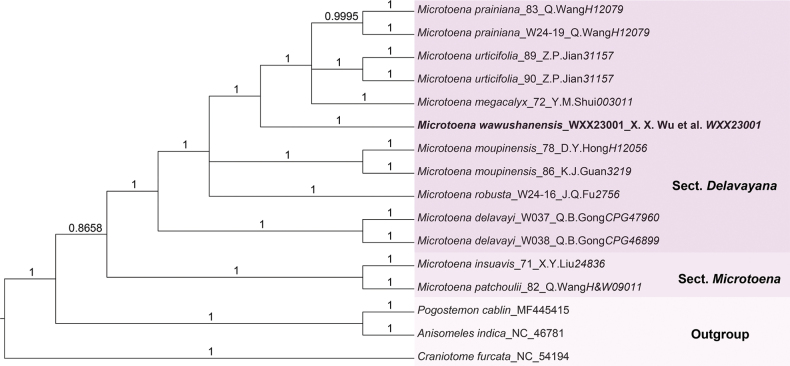
Phylogenetic placement of *Microtoenawawushanensis* sp. nov. within the *Microtoena* inferred by Bayesian Inference (BI), based on 81 coding regions (dataset CP81).

**Figure 2.**
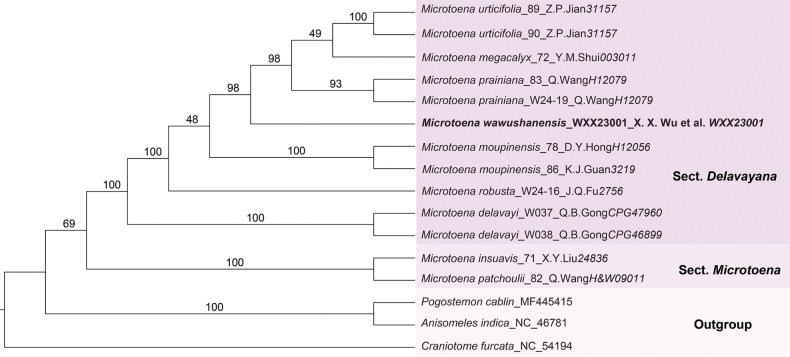
Phylogenetic placement of *Microtoenawawushanensis* sp. nov. within the *Microtoena* inferred by Maximum Likelihood (ML), based on 81 coding regions (dataset CP81).

The phylogenetic tree inferred from Bayesian Inference (BI) methods and Maximum Likelihood (ML) analyses identified the genus *Microtoena* as monophyletic (BI-PP = 0.86, ML-BS = 69%) (Figs 1, 2), consistent with findings from previous studies (Wang 2018). The topologies of the BI and ML phylogenetic trees are largely congruent, with *M.wawushanensis* clustering within sect. Delavayana. Within this section, *M.urticifolia* Hemsl., *M.prainiana* and *M.megacalyx* C.Y.Wu formed a clade sister to the potential new species *M.wawushanensis* with high support (BI-PP = 1, ML-BS = 98%). However, the topologies differed between the BI and ML phylogenetic trees in some nodes. The previous clade is sister to *M.moupinensis* (Franch.) Prain and *M.robusta* Hemsl. in BI phylogenetic tree (Fig. 1: BI-PP = 1.00). In the ML tree, the previous clade is sister to *M.moupinensis* with low support (Fig. 2: ML-BS = 48%), then this clade is sister to *M.robusta* (Fig. 2: ML-BS = 100%). *M.robusta*, which shares the characteristic of a white corolla with red markings on the upper lip with the newly-described species. *M.delavayi* Prain, characterised by a pale yellow or yellow corolla with an upper lip that is either densely spotted with purplish-red markings or completely spotless, occupies a basal position within sect. Delavayana, supported by high values (BI-PP = 1.00, ML-BS = 100%). In this study, sect. Microtoena is resolved as a sister clade to sect. Delavayana, comprising *M.patchoulii* (C.B. Clarke ex Hook. f.) C.Y. Wu & S.J. Hsuan and *M.insuavis* with high support (BI-PP = 1.00, ML-BS = 100%) in this study.

### ﻿Morphological comparison

**Figure 3.**
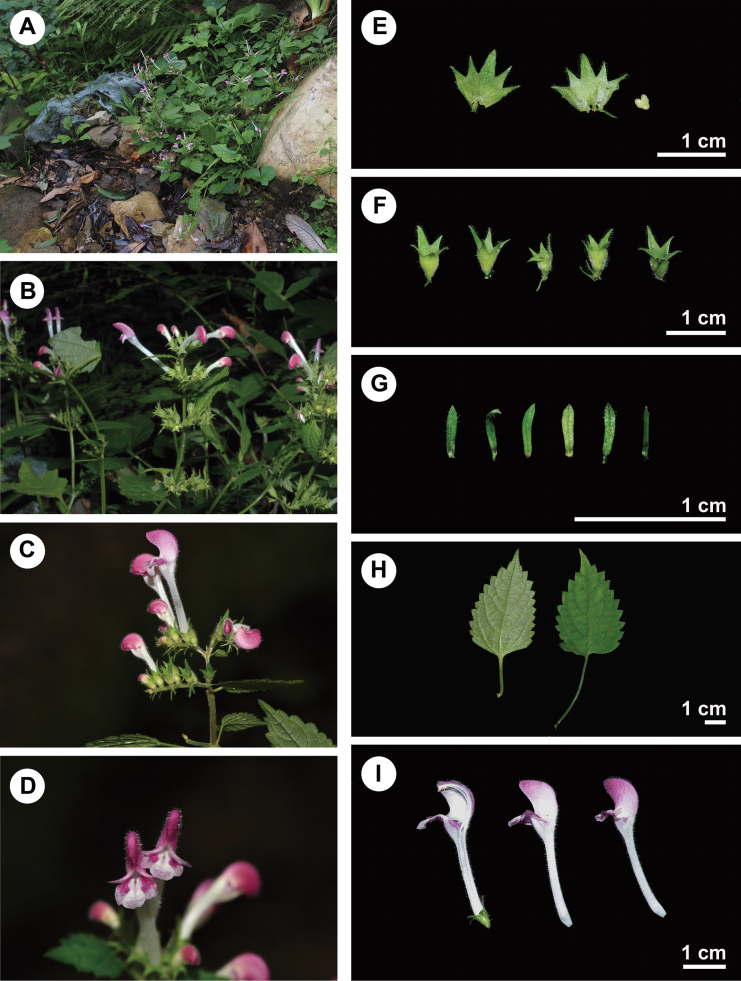
Images of *Microtoenawawushanensis* sp. nov. **A** individual in the flowering period of wild populations **B, C** inflorescence in lateral view **D** lower corolla lip **E** dissected calyxes (outside & inside) **F** calyxes **G** bracts **H** leaves **I** dissected corolla (containing filaments) and corola.

A comprehensive morphological comparison was conducted between the new species and other species of *Microtoena*. Key morphological characteristics of the new species include the inflorescences characterised by lax to more or less compact spike-like panicles (Fig. 3B, C). The corolla is white, marked with red on the upper lip and the lateral lobes of the lower corolla are also red (Fig. 3D). The calyx teeth are triangular-lanceolate to linear-lanceolate or subulate, with the apex typically hooked (Fig. 3E, F). The bracts are linear to lanceolate (Fig. 3G). The leaf base that is cuneate to truncate-subcordate (Fig. 3H). These distinctive traits facilitate the easy differentiation of the new species from all other members of the sect. Delavayana.

The size of the calyx and its teeth has been used to subdivide *Microtoena* (Hsuan 1965; Wu and Li 1977); however, both the calyx and its teeth in this genus continue to grow after anthesis. To address this, we applied three analytical indices for the calyx as proposed by Wang (2018): CLA (calyx length in anthesis), SR (split ratio) and TR (tooth ratio). The CLA value of *M.wawushanensis* was found to range from 0.38 to 0.80 cm, which is similar to *M.moupinensis*. Regarding the SR, this value of *M.wawushanensis* ranged from 0.23 to 0.40, shows variability similar to that of *M.moupinensis*, with a much wider range than the other species (Wang 2018). Concerning the calyx tooth ratio (TR), *M.moupinensis* has five unequal calyx teeth, with one tooth slightly longer than the other four. In comparison, *M.wawushanensis* displays a highly variable and unstable TR (Fig. 3E, F), ranging from 1.36 to 2.13, which is comparable to *M.urticifolia* with a range of 1.83 to 2.24.

The bracts of *Microtoena* are typically linear, lanceolate or ovate and are minute, with some being early deciduous (Wang 2018). Upon measurement, the bracts of *M.wawushanensis* range from 2.10, 11.40 mm in length and 0.40, 0.80 mm in width, with a bract ratio (BR) ranging from 0.13 to 0.18, displaying a linear to lanceolate shape (Fig. 3G). In comparison, the bracts of morphologically similar species such as *M.moupinensis* and *M.urticifolia* are linear, with a BR of approximately 0.10, while *M.prainiana* has ovate bracts, with an average BR of 0.45 and *M.megacalyx* has lanceolate bracts, with an average BR of 0.26.

**Figure 4.**
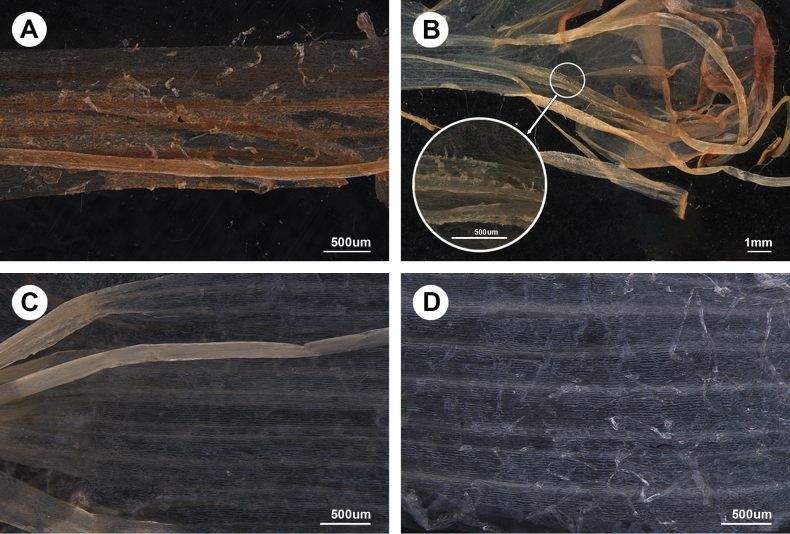
Images of partial filaments morphology of *Microtoenaprainiana* and *M.wawushanensis* sp. nov. **A** the middle part of the filaments of *M.prainiana* (PE 01908066) **B** the upper part of the filaments of *M.wawushanensis***C** the middle part of the filaments of *M.wawushanensis***D** the basal part of the filaments of *M.wawushanensis*.

Morphologically, the new species is characterised by a conspicuously elongated corolla tube (Fig. 3I), indicating its placement within the sect. Delavayana (Wang, 2018). Our observations revealed that the similar species *Microtoenaprainiana* has filaments that are barbate in the middle and lower sections (Fig. 4A), a trait commonly observed within the genus (Wang 2011). In contrast, *M.wawushanensis* exhibits a smooth, glabrous surface, with the hairs being almost imperceptible in the middle region (Fig. 4C), but barbate at both the upper and basal parts of the filaments (Fig. 4B, D).

*Microtoenawawushanensis* exhibits similarities in corolla colouration with two other species in the genus *Microtoena* that have red markings on the upper lip: *M.robusta* and *M.delavayi*. *M.robusta* has a white corolla featuring red markings on the upper lip, similar to the newly-described species, while *M.delavayi* has a pale yellow or yellow corolla and its upper lip may have dense purplish-red markings or be completely devoid of spots. However, *M.robusta* can be easily distinguished from *M.wawushanensis* by its cymes, which are axillary and terminal, dichotomous and lax, as well as by its tiny linear bracts. Likewise, *M.delavayi* is clearly identifiable by its unequal calyx teeth, with one tooth being notably elongated and its characteristic pale yellow or yellow corolla.

Consequently, the combined evidence from morphological and phylogenetic analyses supports the recognition of a new species in *Microtoea*.

### ﻿Taxonomic treatment

#### ﻿ Microtoena wawushanensis

Taxon classificationPlantae

Xue X. Wu & Qiang Wang sp. nov.

A34661C4-8D53-549F-8C8D-ADFB9DE8B006

urn:lsid:ipni.org:names: 77349693-1

Figs 3 , 5 , 6

##### Type.

China • Sichuan Province Meishan City, Hongya County, Wawushan Nature Reserve, growing under the forest by the edge of a riverside, 29°32.2832'N, 102°55.6359'E, 1500 m alt., 14 September 2023, *X. X. Wu et al. WXX23001* (holotype: PE02462560; isotypes: PE02462561, PE02462562, PE02462563, PE02462564, PE02462565).

**Figure 5.**
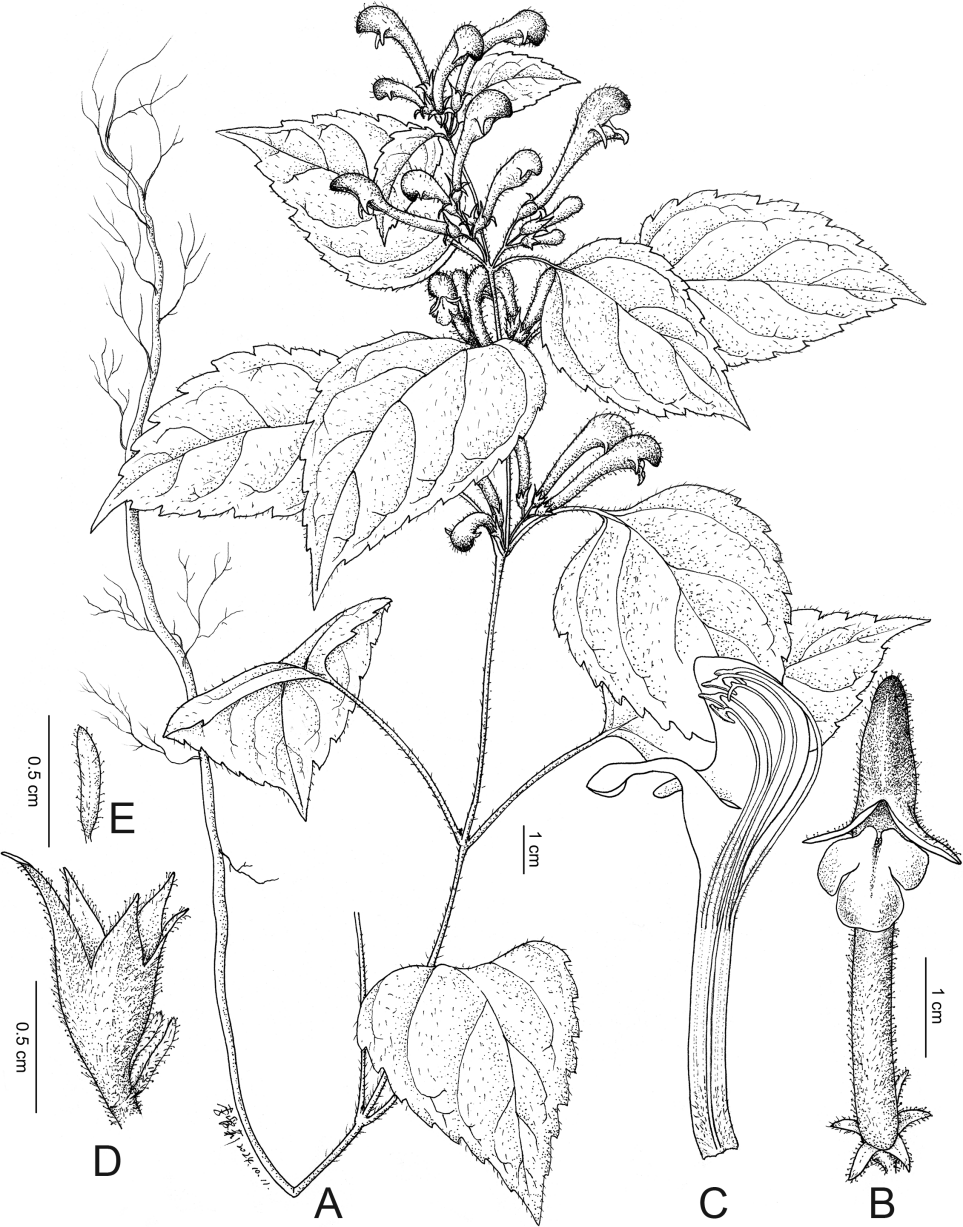
Line drawing of *Microtoenawawushanensis* sp. nov. **A** the whole plant **B** flower (frontal view) **C** dissected corolla **D** calyx with two bracts **E** bract. (Drawn by Ai-Li L).

##### Diagnosis.

*Microtoenawawushanensis* is morphologically similar to *M.moupinensis* (Franch.) Prain and *M.prainiana* Diels (Table 2), but differs from *M.moupinensis* and *M.prainiana* by having a crenate leaf margin with distinct mucrones (vs. with or dentate without any mucro), a cuneate to truncate-subcordate leaf base (vs. truncate-subcordate or cuneate leaf base), a lax, more or less compact to spike-like panicle inflorescence (vs. inflorescence sometimes with one-sided branches or shortly ovoid panicles), linear to lanceolate bracts (vs. linear or ovate bracts), calyx teeth that are triangular-lanceolate, linear-lanceolate to subulate with an apex usually hooked (vs. similar or calyx teeth subulate with a conspicuously hooked apex), a highly variable and unstable calyx tooth ratio: 1.36–2.13 (vs. 1.03–1.49 or five subequal calyx teeth), a white corolla marked with red on both upper lip and the lateral lobes of the lower corolla (vs. yellow or pale yellow corolla) and filaments are barbate at both the upper and basal parts, with the hairs in the middle section being almost imperceptible (vs. filaments are clearly barbate from the lower to middle part).

**Table 2.** Morphological comparison amongst *M.wawushanensis*, *M.moupinensis* and *M.prainiana*.

Morphology	*M.wawushanensis*	*M.moupinensis*	*M.prainiana*
**Leaf margin**	crenate with distinct mucrones	crenate with distinct mucrones	dentate without any mucro
**Leaf base**	cuneate to truncate-subcordat	truncate-subcordate, cuneate, cordate	cuneate
**Inflorescence**	lax, more or less compact, to spike-like panicles	lax, more or less compact, to spike-like panicles, sometimes with 1-sided branches	shortly ovoid panicles
**Bracts**	linear to lanceolate	linear	ovate
**Calyx morphology**	calyx teeth triangular-lanceolate, linear-lanceolate to subulate, with apex usually hooked	calyx teeth triangular-lanceolate, linear-lanceolate to subulate, with apex usually hooked	calyx teeth subulate, apex conspicuously hooked
**Calyx tooth ratio**	highly variable and unstable: 1.36–2.13	variable and unstable: 1.03–1.49	five subequal calyx teeth: 1.1
**Corolla**	corolla white, marked with red on upper lip, the lateral lobes of the lower corolla are marked with red	corolla yellow to pale yellow	corolla uniformly pale yellow
**Stamens**	filaments are barbate at both the upper and basal parts of the corolla tube, with the hairs in the middle section being almost imperceptible	filament barbate on the lower to middle part	filament barbate on the lower to middle part

**Figure 6.**
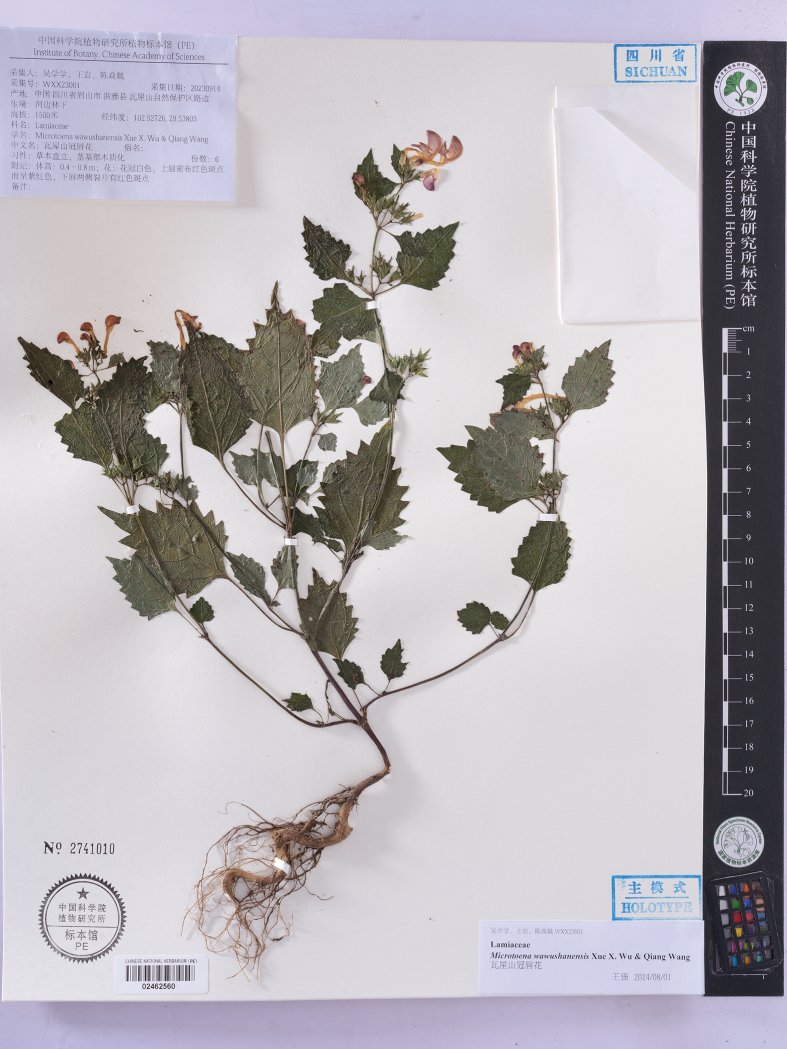
Holotype of *Microtoenawawushanensis* sp. nov. (PE02462560).

##### Description.

Herbs perennial. Stems erect, 0.40–0.80 m tall, base sometimes woody, sparsely puberulent. Leaf petiole 3–9 cm long; leaf blade ovate to oblong-ovate, triangular-ovate, 3.91–9.86 cm long, 3.91–9.86 cm broad, sparse hairs on the adaxial surface, with few hairs visible only along the veins on the abaxial surface and the rest of the abaxial surface glabrous; base truncate or cuneate; margin coarsely serrate to mucronate crenate, with distinct mucrones; apex acuminate to shortly caudate-acuminate. Cymes axillary and terminal, dichotomous, lax, slightly to very compact in spike-like panicles; peduncle inconspicuous. Bracts usually linear to lanceolate, 2.10–11.40 mm long, 0.40–0.80 mm broad. Calyx is 0.38–0.80 cm long at anthesis, densely puberulent, dilated after anthesis and 5-toothed; calyx teeth are triangular-lanceolate, linear-lanceolate to subulate, with the tooth ratio highly variable and unstable and the apex is usually hooked. Corolla white, marked with red on upper lip, 1.76–3.59 cm long, hirsutulous outside; corolla tube conspicuous; upper corolla lip laterally compressed; lower corolla lip 3-lobed, middle lobe subcircular, wider than lateral lobes, lateral lobes marked with red. Stamens 4, filament barbate at the upper and basal parts, while the middle section has nearly imperceptible hairs. Nutlets dark brown to black, smooth.

##### Distribution and habitat.

Currently, *M.wawushanensis* has been found in Wawushan Nature Reserve, Hongya County, Meishan City, Sichuan Province, China. It occurs by the edge of a riverside with weak light, at an elevation of 1500 m. In the type locality, the companion species mainly include *Bistortaamplexicaulis* (D. Don) Greene, *Urticafissa* E. Pritz., *Lecanthuspeduncularis* (Wall. ex Royle) Wedd., *Cyathulaofficinalis* K. C. Kuan, *Sinacaliadavidii* (Franch.) Koyama and *Stachyuruschinensis* Franch.

##### Phenology.

Flowering from August to September, fruiting in September.

##### Etymology.

The specific epithet is derived from the type locality of the new species, i.e. the Wawushan Nature Reserve in southwest Sichuan Province, China and the Latin suffix-*ensis*, indicating the place of origin or growth.

##### Vernacular name.

(assigned here). Simplified Chinese: 瓦屋山冠唇花 (Chinese pinyin: wǎ wū shān guàn chún huā).

##### Conservation assessment.

The ongoing field investigation has identified only one population of this taxon that is endemic to the Wawushan Nature Reserve. Additional fieldwork is necessary to gain a better understanding of this species. According to the guidelines of the IUCN Red List Categories and Criteria (IUCN Standards and Petitions Committee 2022), *Microtoenawawushanensis* is assessed as data deficient (DD).

## Supplementary Material

XML Treatment for Microtoena wawushanensis

## ﻿Additional information

### Author ORCIDs

Xue-Xue Wu https://orcid.org/0009-0006-8645-4100

Yan Wang https://orcid.org/0000-0002-8818-0282

Yan-Yi Chen https://orcid.org/0009-0008-5864-8121

Qiang Wang https://orcid.org/0000-0002-0699-2034

### Data availability

All of the data that support the findings of this study are deposited at GenBank.

## Supplementary materials

Supplementary material 1 Summary of the quality of samples sequencing data Data type docx https://binary.pensoft.net/file/1209986This dataset is made available under the Open Database License (http://opendatacommons.org/licenses/odbl/1.0/). The Open Database License (ODbL) is a license agreement intended to allow users to freely share, modify, and use this Dataset while maintaining this same freedom for others, provided that the original source and author(s) are credited.Xue-Xue Wu, Yan Wang, Yan-Yi Chen, Qiang Wang
