# Supplementary material for: ﻿Microtoenawawushanensis (Lamiaceae, Lamioideae): A new species from Sichuan, China
Source: PhytoKeys. 2024 Dec 30;250:223–36. doi: 10.3897/phytokeys.250.139362 (PMC11704741; doi:10.3897/phytokeys.250.139362)
Supplement: Supplementary material 1 — Summary of the quality of samples sequencing data [file phytokeys-250-223_article-139362__-s001.docx]

**Table S1**. Summary of the quality of samples sequencing data

| **Sample Name** | **Library Name** | **Raw Reads** | **Clean Reads** | **Raw Base(G)** | **Clean Base(G)** | **Effective(%)** | **Error(%)** | **Q20(%)** | **Q30(%)** | **GC(%)** |
| --- | --- | --- | --- | --- | --- | --- | --- | --- | --- | --- |
| 71 | FDSW230177890-1a | 1041212352 | 826959644 | 156.18 | 124.04 | 79.42 | 0.02 | 98.36 | 95.62 | 40.51 |
| 72 | FDSW230177891-1a | 1016292618 | 938046178 | 152.44 | 140.71 | 92.3 | 0.02 | 98.24 | 95.14 | 37.38 |
| 78 | FDSW230177897-1b | 502148868 | 499855398 | 75.32 | 74.98 | 99.54 | 0.06 | 96.24 | 87.95 | 58.04 |
| 82 | FDSW230177901-1a | 350657626 | 346017208 | 52.6 | 51.9 | 98.68 | 0.05 | 97.47 | 91.53 | 34.54 |
| 83 | FDSW230177902-1a | 307236158 | 304719266 | 46.09 | 45.71 | 99.18 | 0.06 | 96.41 | 88.7 | 47.12 |
| 86 | FDSW230177905-1a | 606387986 | 459121278 | 90.96 | 68.87 | 75.71 | 0.04 | 98.79 | 96.13 | 41.03 |
| 89 | FDSW230177908-1a | 517406812 | 480069314 | 77.61 | 72.01 | 92.78 | 0.04 | 98.24 | 94.07 | 40.93 |
| 90 | FDSW230177909-1a | 280832266 | 247658530 | 42.12 | 37.15 | 88.19 | 0.04 | 98.31 | 94.52 | 38.2 |
| WXX23001 | FDSW230308636-1a | 366256508 | 364596036 | 54.94 | 54.69 | 99.55 | 0.04 | 98.43 | 95.6 | 42.23 |
| W24-16 | FDSW240323137-1r | 543921028 | 306204742 | 81.59 | 45.93 | 56.3 | 0.02 | 95.51 | 89.23 | 37.44 |
| W24-19 | FDSW240323140-1r | 348166944 | 332551188 | 52.23 | 49.88 | 95.51 | 0.01 | 96.89 | 91.82 | 45.39 |
| W037 | FDSW230407586-1r | 423429470 | 421939140 | 63.51 | 63.29 | 99.65 | 0.03 | 96.25 | 90.05 | 40.53 |
| W038 | FDSW230407587-1r | 623942896 | 621378724 | 93.59 | 93.21 | 99.59 | 0.03 | 96.19 | 89.8 | 38.32 |
